# Validity and Reliability of the Secondary Traumatic Stress Scale—Chinese Version

**DOI:** 10.3389/fsurg.2022.882712

**Published:** 2022-04-01

**Authors:** Yi He, Zhiqun Liu, Juan Zhang, Jiapei Yao, Huan Xiao, Huan Wan

**Affiliations:** ^1^Department of Otolaryngology Head and Neck Surgery, Hunan Provincial People's Hospital (The First Affiliated Hospital of Hunan Normal University), Changsha, China; ^2^Department of Respiratory Therapy, Hunan Provincial People's Hospital (The First Affiliated Hospital of Hunan Normal University), Changsha, China; ^3^Department of Biliary Surgery, Hunan Provincial People's Hospital (The First Affiliated Hospital of Hunan Normal University), Changsha, China; ^4^Department of Orthopedics, The Affiliated Changzhou No.2 People's Hospital of Nanjing Medical University, Changzhou, China; ^5^Department of Nursing, Hunan Provincial People's Hospital (The First Affiliated Hospital of Hunan Normal University), Changsha, China

**Keywords:** secondary traumatic stress, validity, reliability, scale, Chinesization

## Abstract

**Objectives:**

To test the validity and reliability of the Secondary Traumatic Stress Scale—Chinese version in clinical nurses.

**Methods:**

According to the translation principles of the Brislin Scale, the original scale was translated, back translated and cross-culturally adapted to form the Chinese version of the Secondary Traumatic Stress Scale. Nurses in three general hospitals in Changsha, Hunan province were surveyed by convenient sampling method from July 2020 to September 2021. Exploratory factor analysis, confirmatory factor analysis, content validity and criterion validity was used to evaluate the validity of the scale. Internal consistency Cronbach's α coefficient, split-half reliability and test-retest reliability were used to evaluate the reliability of the scale.

**Results:**

A total of 678 nurses were included in the study. There were 460 people in sample 1 and 218 people in sample 2. Two common factors were extracted by exploratory factor analysis. The cumulative contribution was 65.560%. The two-factor structure model was good (χ^2^/df = 3.137, CFI = 0.928, IFI = 0.929, GFI = 0.842, TLI = 0.917, RMSEA = 0.099). The I-CVI of the scale was 0.8–1.0. The S-CVI/Ave was 0.94. The Cronbach's alpha coefficient is 0.956. The broken half reliability is 0.920. The retest reliability is 0.910.

**Conclusion:**

This study identified two components of the Secondary Traumatic Stress Scale—Chinese version, which has 2 dimensions and 17 items. With good validity and reliability, it is suitable for the assessment of secondary traumatic stress among clinical nurses in the Chinese context.

## Introduction

Secondary traumatic stress (STS) is a pattern of psychological symptoms in which the helper exhibits disturbing or painful psychological symptoms during or after helping without directly experiencing a traumatic event (1). STS was originally described as Compassion fatigue (CF) (2). But it does not include the concept of empathy. They should therefore be defined and measured differently (3, 4). Symptoms of STS include exhaustion, hyperarousal, avoidance and numbness. Similar to Post-traumatic stress disorder (PTSD) (5). The difference is that PTSD occurs in individuals who have direct exposure to traumatic events (6), whereas STS occurs in professionals who have indirect exposure to traumatic individuals (1). At the beginning, STS had some similarities with PTSD and CF. Researchers often assess STS using measures of PTSD or the Occupational Quality of Life Scale (ProQOL) (7). Neither was specifically designed for that purpose. The Secondary Traumatic Stress Scale (STSS) (1) was compiled by Bride BE in 2004 according to the concept of STS proposed by Figley. It measures intrusion, avoidance, and arousal symptoms triggered by indirect exposure to traumatic events. Its psychometric properties have been validated (8). Over the past decade, STSS has become the standard tool for assessing STS. It has been used by doctors, nurses, midwives, respiratory therapists, mental health workers and other social workers in many regions (9–12). It has been translated into Hungarian (13), French (14), German (15), Japanese (16) and other languages.

The stimulation induced by STS was short in duration. STS can suddenly occur without much warning (17). The incidence of STS may be high for frontline mental health professionals and social workers (including nurses, mental psychiatrists, first responders, and victim advocates, etc.) (5, 18, 19). Because they frequently work with victims of all forms of trauma, such as listening to trauma victims describe the physical and psychological horrors they have experienced, providing a variety of services including emotional support, in-person counseling and psychological education information. As a key member of the medical treatment team, nurses are usually the first people to contact patients and their families. Nurses are also the main personnel to observe patients' pain and distress symptoms. Their professional clinical competence is the basic requirement to provide safe and effective care for patients. A large number of studies have shown that STS is common among nurses (20, 21). STS can have a wide range of impacts on personal and professional life, including various somatization symptoms, sleep disorders, depression, anxiety, interpersonal relationship damage and other aspects (22). Most of them are negative, which may affect the ability level and nursing quality of nurses (23). This will affect patient satisfaction and even the stable development of the hospital. Improving the mental health of nursing staff can effectively improve the nursing level (24). Therefore, it is necessary to assess the STS of clinical nurses effectively.

Although the compassion fatigue scale has been sinicized and put into use by scholars. Zakeri (25) pointed out that we should study STS and compassion fatigue as independent but related structures. It also focuses on understanding the factors related to STS in nurses. So as to help them reduce STS. Currently, there is a lack of assessment tools to directly measure STS among nursing staff in China, which is not conducive to understanding the current situation of STS among nursing staff in China, nor to promoting corresponding social support systems and using positive coping mechanisms to reduce STS. Given the potential harmful effects of STS and the fact that effective interventions depend on accurate assessment. The introduction of STSS can help to quickly identify the mental state of nursing staff. To provide a basis for timely and accurate targeted interventions. Therefore, this study aims to sinicize STSS and evaluate the validity and reliability of the Chinese version of STSS (C-STSS) in nursing staff. To provide a reliable tool for assessing STS of clinical nursing staff.

## Methods

### Participants

The participants included 678 clinical nurses from three general hospitals in Changsha, Hunan province (Hunan Provincial People's Hospital, Hunan Provincial Second People's Hospital, Changsha Xingsha Hospital). All of them had worked for more than one year.

Sample 1: A total of 460 clinical nurses were included.
Sample 2: A total of 218 clinical nurses were included.

The proposal of this research was approved by the ethics committee of Hunan Provincial People's Hospital (The first affiliated hospital of Hunan normal university). All subjects gave informed consent to this study.

### Measures

#### Secondary Traumatic Stress (STSS)

The scale is a self-rating scale. The frequency of STS-related symptoms in the previous 7 days was assessed. It consists of three subscales of intrusion, avoidance and arousal, with 17 items in total. Likert grade 5 scoring method was adopted. The higher the score was, the higher the frequency of symptoms appeared. A total score below 28 is classified as “no STS”. 28-37 is “mild STS”. 38-43 is “moderate STS”. 44-48 is “high STS”. More than 49 is “severe STS”. The Cronbach's α coefficients of the total scale were 0.94. The α coefficients of the three subscales were 0.83, 0.89 and 0.85 (8).

#### Post-traumatic Stress Disorder Scale Civilian Version (PCL-C)

There are 17 items in this scale. It includes three dimensions: reexperience, avoidance and over-arousal. To assess individual PTSD symptoms and their severity. Likert 5-level scoring method is adopted, with a total score of 17-85. The higher the score, the more severe the PTSD degree (26). The Cronbach's α coefficient of the scale in this study was 0.956.

#### Chinese Version of the Compassion Fatigue Scale (C-CFS)

A total of 30 items. It is mainly used for measuring the clinical nurses in satisfaction, traumatic stress, job burnout, intrusive thoughts and fears of five aspects. Likert grade 5 scoring method was adopted. Score 1-5 points from “none” to “always”. A few items are scored backwards. The higher the score prompt more sympathy for the degree of fatigue (27). The Cronbach's α coefficient of the scale in this study was 0.930.

### Procedures

Translating-back translation scale using Brislin's intercultural translation model. Firstly, two bilingual experts were invited to translate STSS. One was a doctor of nursing who was familiar with psychological terms. The other was a non-medical person. Then, the research team members compared and merged the Chinese versions translated by the two experts. The differences were discussed with two experts. Form proofread version STSS. Then, a nursing master candidate with overseas study experience and a bilingual expert were invited to translate the Chinese proofread version of STSS back into English. In order to ensure the quality of translation, they were not informed that they were doing translation work. A bilingual expert and research team members will compare and discuss the translated version with the original version to form the first version of STSS.

In order to ensure the pertinence and effectiveness of the scale after Chinese version, five experts were invited to form a cultural debugging group (doctor of psychology, master of psychology and doctor of nursing) through face-to-face discussion or E-mail to debug the preliminary STSS. Comments and suggestions were put forward based on whether the meaning of each item in the questionnaire was clear, whether the language expression was simple, accurate, direct and easy to understand. Then the items were revised and integrated again.

The convenience sampling method was used to conduct a preliminary survey in June 2020. 40 clinical nurses who met the inclusion and exclusion criteria were selected for the preliminary experiment. The internal consistency reliability of the C-STSS was calculated according to the results of the preliminary experiment. The results showed that all 40 clinical nurses could understand the meaning of each item in the scale. The average time to complete the questionnaire was 5-10min. The analysis of the investigation results of the 40 subjects showed that the Cronbach's α coefficient of the C-STSS was 0.943.

### Data Collection

The convenience sampling method was used to investigate the clinical nursing staff in three general hospitals by using questionnaire star in two stages. Before data collection, use uniform standard guidance language. It is stipulated that the questionnaire can be submitted only after all items have been answered to avoid missing selection. The same client can only submit once. In the first stage, 460 valid questionnaires (sample 1) were collected. 218 valid questionnaires (sample 2) were collected in the second stage. A total of 678 questionnaires were collected.

### Statistical Methods

In this study, frequency and percentage were used to describe the general data of the research object. SPSS 26.0 was used for exploratory factor analysis of sample 1 (n = 460). The content validity, internal consistency reliability, half-fold reliability and retest reliability of the scale were evaluated. AMOS 21.0 was used for confirmatory factor analysis and criterion validity analysis of sample 2 (n = 218). Test level α = 0.05.

## Results

### Demographics

Table 1 displays the demographics of both samples. A total of 460 clinical nurses were included in Sample 1. 15 cases were male (3.3%). Female 445 cases (96.7%). A total of 218 clinical nurses were included in Sample 2. 17 cases were male (7.8%). Female 201 cases (92.2%).

**Table 1:** The personal and demographic information of the participating in the study.

**Sample 1**	**Variable**	**Number (*n*)**	**Percentage (%)**	**Sample 2**	**Variable**	**Number (*n*)**	**Percentage (%)**
Age	<25y old	117	25.4	Age	<25y old	24	11.0
	25–30y old	143	31.1		25–30y old	66	30.3
	30–40y old	157	34.1		30–40y old	113	51.8
	>40y old	43	9.3		>40y old	15	6.9
Gender	Male	15	3.3	Gender	Male	17	7.8
	Female	445	96.7		Female	201	92.2
Type of education	High school degree	100	21.7	Type of education	High school degree	19	8.7
	Bachelor degree	351	76.3		Bachelor degree	185	84.9
	Master degree or above	9	2.0		Master degree or above	14	6.4
Professional qualification	Primary	300	65.2	Professional qualification	Primary	123	56.4
	Intermediate	135	29.3		Intermediate	83	38.1
	Senior	25	5.4		Senior	12	5.5

### Validity

#### Structural Validity

##### Exploratory Factor Analysis

The results of Bartlett's sphericity test showed that the KMO value was 0.968. The Bartlett's sphericity test χ^2^ value was 5,767.238. The degree of freedom was 136 (*P* < 0.001). It suggests that the data in this study are suitable for factor analysis. Principal component analysis (PCA) was used to extract factors with characteristic roots >1 without limiting the number of factors. Run a lithograph at the same time. Two common factors were extracted, with a cumulative contribution rate of 65.560%. The maximum variance orthogonal rotation method was adopted for exploratory factor analysis. The factor load matrix was rotated. There is no item with factor load < 0.40 in the corresponding common factor. And no items with a common degree < 0.2. Therefore, the questionnaire with 17 items and 2 common factors was finally formed. The cumulative variance contribution rate was 65.560% (Table 2). Factors are defined according to the items and meanings contained in each common factor. Factor 1 stress (Including items 1, 4, 5, 7, 8, 9, 11, 15, 16, 17. A total of 10 items. Cronbach's α 0.718). Factor 2 invasion and avoidance (Including items 2, 3, 6, 10, 12, 13, 14. A total of 7 items. Cronbach's α 0.696).

**Table 2:** C-STSS exploratory factor analysis results (*n* = 460).

**Item**	**$\bar{x}$ ±SD**	**Factor 1**	**Factor 2**	**Common degrees**
I was less active than usual.	3.18 ± 1.007	0.799		0.690
I felt discouraged about the future.	2.79 ± 1.024	0.780		0.721
I had little interest in being around others.	2.64 ± 1.025	0.746		0.686
I had trouble concentrating.	2.70 ± 0.941	0.746		0.712
I felt jumpy.	2.86 ± 0.996	0.720		0.710
I felt emotionally numb.	2.66 ± 0.977	0.679		0.622
I expected something bad to happen.	2.55 ± 0.974	0.630		0.702
I had trouble sleeping.	2.93 ± 1.052	0.617		0.457
I was easily annoyed.	2.73 ± 0.992	0.601		0.600
I noticed gaps in my memory about patient sessions.	2.63 ± 0.920	0.552		0.397
I wanted to avoid working with some patients.	2.31 ± 0.959		0.813	0.767
I avoided people, places, or things that reminded me of my work with patients.	2.36 ± 0.952		0.756	0.721
I had disturbing dreams about my work with patients.	2.16 ± 0.953		0.745	0.667
My heart started pounding when I thought about my work with patients.	2.61 ± 0.998		0.737	0.623
It seemed as if I was reliving the trauma(s) experienced by my patient(s).	2.35 ± 0.997		0.733	0.650
Reminders of my work with patients upsets me.	2.50 ± 0.955		0.733	0.738
I thought about my work with patients when I didn't intend to.	2.66 ± 0.928		0.716	0.682
Eigenvalue		5.717	5.429	
Explanatory variance percentage		33.627	31.933	
Cumulative contribution rate				65.560

##### Confirmatory Factor Analysis

AMOS was used to perform confirmatory factor analysis on the relevant data of sample 2 (*n* = 218). The fitting indexes of the model were as follows. The χ^2^/ DF was 3.137. The CFI was 0.928. The GFI was 0.842. The IFI was 0.929. The TLI was 0.917. The RMR was 0.048. The RMSEA was 0.099.

#### Content Validity

In this study, 10 experts from psychology related fields were invited to form an expert group to score the scale with level 4 Content Validity Index (CVI). All the experts had been engaged in psychology-related treatment and nursing or research for more than 5 years. Members of the expert panel evaluated each item of the questionnaire. Finally calculated the Item-Content Validity Index (I-CVI) of the C-STSS. I-CVI ranged from 0.80 to 1.00. The scale-content Validity Index (S-CVI) was 0.94. The results are shown in Table 3.

**Table 3:** Content validity of C-STSS (*n* = 17).

**Item**	**I-CVI**
I felt emotionally numb.	0.9
My heart started pounding when I thought about my work with patients.	0.9
It seemed as if I was reliving the trauma(s) experienced by my patient(s).	0.8
I had trouble sleeping.	0.8
I felt discouraged about the future.	1.0
Reminders of my work with patients upsets me.	1.0
I had little interest in being around others.	0.9
I felt jumpy.	1.0
I was less active than usual.	1.0
I thought about my work with patients when I didn't intend to.	0.9
I had trouble concentrating.	0.9
I avoided people, places, or things that reminded me of my work with patients.	1.0
I had disturbing dreams about my work with patients.	1.0
I wanted to avoid working with some patients.	1.0
I was easily annoyed.	1.0
I expected something bad to happen.	0.9
I noticed gaps in my memory about patient sessions.	0.9
S-CVI/Ave	0.94

#### Criterion Correlation Validity

PCL-C and C-CFS are used as calibration tools of C-STSS. The total score of STSS and its two factors (stress, invasion and avoidance) were positively correlated with the total score of PCL-C and its subscales (re-experience, avoidance and over-arousal) (*P* < 0.01). It was positively correlated with the total score of C-CFS and its four factors (traumatic stress, job burnout, intrusive thinking, fear) (*P* < 0.01) (Table 4).

**Table 4:** Correlation between C-STSS score and criteria [r, (*n* = 218)].

**Scale**	**C-STSS score**	**Stress**	**Invasion and evasion**
**PCL-C**	0.881^**^	0.875^**^	0.800^**^
Re-experience	0.767^**^	0.724^**^	0.750^**^
Avoidance	0.828^**^	0.809^**^	0.772^**^
Over-arousal	0.799^**^	0.849^**^	0.647^**^
**C-CFS**	0.629^**^	0.580^**^	0.635^**^
Satisfaction	0.061	0.077	0.033
Traumatic stress	0.709^**^	0.620^**^	0.766^**^
Job burnout	0.729^**^	0.682^**^	0.723^**^
Intrusive thinking	0.704^**^	0.605^**^	0.774^**^
Fear	0.661^**^	0.666^**^	0.587^**^

** *P < 0.01*.

#### Reliability

The results of this study show that the Cronbach's α coefficient of C-STSS is 0.956. The Cronbach's α coefficient of stress dimension is 0.931. The Cronbach's α coefficient of invasion and avoidance dimension is 0.926. All were >0.70. The half-reliability of the C-STSS is 0.934. The half-reliability of the two dimensions is 0.920. Forty nurses were selected for retest at intervals of 2 weeks for convenience. The retest reliability of The C-STSS was 0.910. The retest reliability of the two dimensions was 0.753 and 0.888 respectively.

## Discussion

With the transformation of biology-psychology-social medicine model, strengthening the construction of mental health service system and standardization of management related policies. The concern for the mental state of clinical medical workers has become a widespread concern in the society (28). As the susceptible population of STS, the inappropriate psychological stress of nursing staff not only affects their physical and mental health, but also affects the quality of nursing service (29, 30). Therefore, it is particularly important to select appropriate and reliable assessment tools for early identification of related symptoms. The purpose of this study is to provide a reliable tool for the evaluation of STS for clinical nurses by sinicizing STSS and testing the reliability and validity of the application of STSS in clinical nurses.

Validity refers to the accuracy, validity and correctness of the measurement content of an evaluation scale. Including structure validity and content validity (31). Exploratory factor analysis was used to verify the structural validity of the C-STSS in this study. Principal component analysis was used to extract the common factor and generate the gravel map of the factor structure. The item could be considered to be in the factor when the factor load value and common degree ≥0.40. The exploratory factor analysis results of this study showed that the C-STSS extracted two common factors and retained all the items in the original scale, with a cumulative contribution rate of 65.560%. Finally, a questionnaire with 2 dimensions and 17 items was formed. One less than the three dimensions in the original scale. The item distribution was not completely consistent with the original scale. The reasons may be as follows: ①regional cultural differences; ②differences in research objects, such as gender, age, position, economic status, etc.; ③time difference. By careful analysis of the items contained in the two dimensions of C-STSS, it can be found that the first dimension—stress dimension (items 1, 4, 5, 7, 8, 9, 11, 15, 16, 17) mainly describes the influence of STS on helpers' daily life and psychological state (including sleep, mood, concentration, enthusiasm, etc.). And dimension 2—invasion and avoidance dimension (items 2, 3, 6, 10, 12, 13, 14) mainly describe the content related to the trauma victims they help.

In order to further confirm the rationality of this dimension division, this study conducted an in-depth analysis of the dimensions of the scale and the distribution of items contained in it. The results showed that the validity of all dimensions and the overall validity of the C-STSS was good. The I-CVI of The C-STSS ranged from 0.80 to 1.00. Both >0.80. The S-CVI/Ave was 0.94. Greater than 0.90. This indicates that each item of the scale had high content validity. The results of internal correlation analysis showed that the correlation coefficient between each item of the scale and the total score of the scale was 0.616–0.834. The correlation coefficient between stress dimension and the total table was 0.965. The correlation coefficient between invasion and avoidance dimension and the total table was 0.932. The correlation coefficient between the two dimensions was 0.806. All of them were statistically significant (*P* < 0.001). This shows that the internal consistency of the scale is good. In addition, the correlation coefficients between the two dimensions and the total table are higher than those between the two dimensions, indicating that each dimension is consistent with the overall concept and relatively independent. Finally, according to the fitting results of the model by confirmatory factor analysis, it can be seen that all fitting indexes reach the ideal standard. The model fits well.

Reliability is to evaluate the stability, equality and internal consistency of the results measured by the scale. Including internal and external reliability. The greater the reliability of the scale, the smaller the standard error of measurement (32). The internal consistency reflects the internal reliability among the measurement items. It checks whether each item of the scale measures the same content. The results of this study show that the Cronbach's α coefficient of The C-STSS is 0.956. The Cronbach's α coefficient of stress dimension is 0.931. The Cronbach's α coefficient of invasion and avoidance dimension is 0.926. All >0.70. Indicating that the C-STSS has good internal consistency. The half-fold reliability of C-STSS is 0.934. The half-fold reliability of both dimensions is 0.920. Indicating that the scale has good internal relevance. The most commonly used evaluation index of external reliability is retest reliability. The retest reliability of The C-STSS is 0.910, >0.80. The retest reliability of the 2 dimensions is 0.753 and 0.888, which indicating that the scale has good cross-time stability. Therefore, the C-STSS has good internal and external reliability and is reliable.

## Conclusions

Due to specific occupational factors, long-term exposure of nurses to stressors may result in job dissatisfaction or burnout. Especially in the context of the COVID-19 epidemic in the past 2 years, their work intensity and psychological stress often exceed load. The psychological problems of medical workers have become a topic of general concern. The psychosomatic health of nursing staff needs more attention and intervention. The C-STSS is an easy-to-implement scale with 17 items. It has a high level of internal consistency reliability and validity. All the evaluation indexes of the scale meet the requirements of measurement, which can be used for the evaluation of STS of clinical medical staff. The application of this scale in China has certain significance.

### Limitations

The sampling sites in this study are only in Changsha city of Hunan Province. It doesn't represent all caregivers in the country. The sample range can be expanded in the future to further verify its applicability. In addition, the subjects were all nursing staff. Cannot represent other social workers such as: mental health professionals, child protection workers, etc. The scale can be further expanded by studying other social workers.

## Data Availability Statement

The raw data supporting the conclusions of this article will be made available by the authors, without undue reservation.

## Ethics Statement

The studies involving human participants were reviewed and approved by Hunan Provincial People's Hospital (The First Affiliated Hospital of Hunan Normal University). The patients/participants provided their written informed consent to participate in this study.

## Author Contributions

YH, ZL, and JZ conceived the study concept and designed the study. HW and HX participated in data collection. YH and JY analyzed the data. YH wrote the initial draft of the manuscript and corrected the manuscript. All the authors read and approved the final version of the article.

## Conflict of Interest

The authors declare that the research was conducted in the absence of any commercial or financial relationships that could be construed as a potential conflict of interest.

## Publisher's Note

All claims expressed in this article are solely those of the authors and do not necessarily represent those of their affiliated organizations, or those of the publisher, the editors and the reviewers. Any product that may be evaluated in this article, or claim that may be made by its manufacturer, is not guaranteed or endorsed by the publisher.
